# The role and challenges of the food industry in addressing chronic disease

**DOI:** 10.1186/1744-8603-6-10

**Published:** 2010-05-28

**Authors:** Derek Yach, Mehmood Khan, Dondeena Bradley, Rob Hargrove, Stephen Kehoe, George Mensah

**Affiliations:** 1 Senior Vice President of Global Health Policy, PepsiCo, 700 Anderson Hill Road, Purchase, New York, USA; 2 Chief Scientific Officer, PepsiCo, 700 Anderson Hill Road, Purchase, New York, USA; 3 Vice President of Global Nutrition, PepsiCo, 700 Anderson Hill Road, Purchase, New York, USA; 4 Vice President of Research and Development (Europe), PepsiCo, Walkers Snack Foods Bursom Road, Beaumont Leys, Leicester, LE4 1BS, UK; 5 Vice President of Public and Government Affairs, PepsiCo and Co-Chairman International Food and Beverage Alliance, 700 Anderson Hill Road, Purchase, New York, USA; 6 Director of Heart Health and Global Health Policy, PepsiCo, 700 Anderson Hill Road, Purchase, New York, USA

## Abstract

**Summary:**

Increasingly, food companies play an important role in stemming the rising burden of nutrition-related chronic diseases. Concrete actions taken by these companies include global public commitments to address food reformulation, consumer information, responsible marketing, promotion of healthy lifestyles, and public-private partnerships. These actions are reviewed together with eleven specific PepsiCo goals and commitments that address products, the marketplace, and communities at large. Interim progress on these goals and commitments are discussed as well as constraints hampering faster progress. Further disease prevention depends on increasing implementation of private-public initiatives.

## Introduction

The rising global burden of nutrition-related diseases[1] calls for concerted action. There is an emerging urgent need to resolve acute and chronic hunger, malnutrition, and undernutrition[2]. In particular, compromised nutritional status of mothers and children very early in life has significant impact on the future occurrence of chronic diseases such as heart disease and type 2 diabetes[3,4]. Concurrently, populations are faced with increasing implications of overweight and obesity. The World Health Organization (WHO) estimates that in 2005, more than 1 billion people were overweight and 300 million obese, with projections of 1.5 billion people overweight by 2015[1]. Worldwide, 44 percent of diabetes burden, 23 percent of ischemic heart disease burden, and 7 to 41 percent of the burden of some cancers can be attributed to overweight and obesity[5].

This paper reviews the progress that major food manufacturing companies and partnerships are making in nutrition-related health, especially in regard to overweight and obesity. Using PepsiCo as an example of one company, specific steps are outlined that will transform PepsiCo's product portfolio and how nutritional choices are communicated to customers. Also indicated are a number of constraints that hamper more rapid progress. Throughout, it is argued that further progress in chronic disease prevention depends on collaborations across multiple sectors, from agriculture to retailers and private to public, for effective development and distribution of packaged food.

### Food industry responses to calls for action

The food industry has been criticized for contributing to the rise in overweight and obesity[6], yet former WHO Director General, Gro Harlem Brundtland realized that solutions to the overweight and obesity problem depend in part on the innovation and efficiency of the food industry. Brundtland stressed that chronic disease solutions demand private-public collaboration and demonstrated her leadership by hosting the first meeting between CEOs of leading food companies and WHO. In this vein, WHO's *Global Strategy on Diet, Physical Activity and Health *(WHA 57.17) contained several recommendations for the food industry to address specific aspects of chronic disease[7]. Adopted by member states in 2004, the recommendations are summarized in Table 1.

**Table 1:** World Health Organizations Global Strategy on diet, physical activity and health: Food companies' responses and recommendations. *

Specific Recommendation to the Food Industry	Food Industry Response
▪ Promote healthy diets and physical activity in accordance with national guidelines and international standards and the overall aims of the Global Strategy.	▪ Underway through the commitments made by International Food & Beverage Alliance (IFBA) to address the areas of food reformulation, consumer information, responsible marketing, promotion of health lifestyles and public-private partnerships. ▪ IFBA has also established food and beverage industry groups in over 15 countries/regions, including the 27 countries of the European Union and the 6 countries of the Cooperation Council for the Arab States of the Gulf, to allow industry to react according to the different needs and concerns of different Member States rapidly and individually as well as expand company participation at the local and regional level, to optimize the local impact, and ensure that industry efforts take into consideration regional and national differences. ▪ More groups are being established in many more countries.

▪ Limit the levels of saturated fats, *trans*-fatty acids, free sugars, and salt in existing products.	▪ Since the Global Strategy was launched in 2004, the steps taken by the food and beverage industry are very significant and are creating measurable improvements showing a major reduction in the marketing of products high in fat, sugar and salt to children less than12 years of age. ▪ IFBA companies have reformulated and/or introduced over 28,000 nutritionally enhanced products globally. This includes specifically reducing or eliminating trans-fat in about 18,000 products. Calories were reduced and saturated fats, sugar, carbohydrates, and sodium were also eliminated or reduced in a significant number of products. At the same time, many products were fortified with vitamins, minerals, whole grains and/or fiber. ▪ IFBA members are also developing product formulations that compensate for chronic micronutrient shortages sometimes found in the developing world--countries in which chronic shortages of iron, vitamin A and iodine in particular can have far-reaching health consequences.

▪ Continue to develop and provide affordable, healthy and nutritious choices to consumers.	▪ See above in addition, not IFBA members have increased investments in R&D aimed at achieving this and each of the companies employ scientists, nutritionists and engineers to develop innovative foods and beverages, and have established processes for internal and external expert and scientific review of their nutrition standards which are then used to drive product innovation.

▪ Provide consumers with adequate and understandable product and nutrition information.	▪ Ongoing efforts continue, but require closer government oversight and interaction to have impact. ▪ IFBA companies have also increased the use of consumer information tools, including websites, help lines, in-store leaflets, and brochures.
▪ Practice responsible marketing that supports the Strategy, particularly with regard to the promotion and marketing of foods high in saturated fats, *trans*-fatty acids, free sugars, or salt, especially to children.	▪ Considerable progress has been made through the IFBA pledge, which is being implemented globally and is subject to external audit. ▪ IFBA companies' engaged Accenture to provide a global "snapshot" of companies' compliance with their marketing commitments. Accenture tested compliance in 12 markets around the world. Accenture reported a 98.17% compliance rate for TV advertising, 100% compliance for print advertising and found only one instance of non-compliance on the internet.

▪ Issue simple, clear and consistent food labels and evidence-based health claims that will help consumers to make informed and healthy choices with respect to the nutritional value of foods.	▪ Requires clarity from WHO on optimal way forward. Many individual company efforts are underway. ▪ Many IFBA companies have improved the labeling on their packaging to provide easily-understandable nutritional information, including guideline daily amounts (GDAs) or Daily Value, ingredient listings, and key nutrients. IFBA companies have also made significant progress in implementing full nutritional labeling on a voluntary basis where full nutritional labeling is not compulsory. ▪ For example, 88% of companies surveyed in Kenya, South Africa, and Uganda are already exceeding the legal labeling requirements and 75% plan on adding more nutritional labeling in the next 24 months.

▪ Provide information on food composition to national authorities.	▪ Underway in countries whose governments have clearly stated norms.

*Adapted from WHA 57.17; article 61

The International Food and Beverage Alliance (IFBA) was established in May 2008 to explicitly answer the WHO call to action by formulating a set of five global public commitments. The commitments address: 1) food reformulation; 2) consumer information; 3) responsible marketing; 4) promotion of healthy lifestyles; and 5) public-private partnerships. These goals are signed by the CEOs of top multinational companies - Ferrero, General Mills, Grupo Bimbo, Kellogg's, Kraft Foods, Mars, Nestlé, PepsiCo, The Coca-Cola Company, and Unilever - for which they are now being held accountable by WHO[8]. The significance of the IFBA initiative (and difference from others) lies in two dimensions: scale and reach. In terms of scale, the ten multinational companies account for around 80% of the global advertising spend in the food and beverage industry and collectively have revenues in excess of $350 billion annually. In terms of reach, the multinationals are collectively present in more than 200 countries. The result is the first serious attempt by any stakeholder group to intervene simultaneously on a worldwide basis.

Leading governments are also beginning to address the problem of childhood obesity. In the United States (US), First Lady Michelle Obama launched an obesity initiative, "Let's Move" with the goal to eliminate obesity within a generation. In response, the beverage companies in the US announced that they will enhance the visibility of calorie labeling on products, soda fountains, and vending machines. These efforts complement work underway by food and beverage companies to restrict sales of products high in sugar, salt, and fat in US schools. For example, in 2006 the American Beverage Association (ABA) joined with the Alliance for a Healthier Generation, a joint initiative of the William J. Clinton Foundation and the American Heart Association, to provide School Beverage Guidelines. Shipments of full-calorie soft drinks to schools declined by 95 percent since prior to the implementation of the Guidelines[9]. These US-based initiatives will be adopted and implemented globally by PepsiCo as well as Coca-Cola and the Dr Pepper Snapple Group.

## PepsiCo's Goals and Commitments

Some food companies have independently taken steps to change the nutrition composition of their portfolios. ConAgra Foods announced in October 2009 that it will reduce sodium by an average of 20 percent in roughly 80 percent of its products by the year 2015. In fact, by summer 2011 the company's Chef Boyardee canned pasta will have decreased its sodium by about 35 percent over five years[10]. In March 2010, Kraft Foods also revealed plans to reduce sodium in its North American products by an average of 10 percent by 2012[11].

Shortly after assuming office as the new CEO of PepsiCo, Indra Nooyi announced in 2007 that the company would increasingly address the non-financial aspects of its performance. The phrase "performance with purpose" (PwP) means delivering sustainable growth through investments in the environment, health and nutrition (human sustainability), and the workforce. Larry Thompson, PepsiCo's Legal Counsel has argued that PwP is a business imperative and critical to the long-term growth of companies. He has emphasized the need for corporations to always take the long view in considering their policies and actions[L. Thompson, personal communication, March 21, 2010].

In March 2010, PepsiCo unveiled eleven global goals and commitments summarized in Table 2 that form the core of how PepsiCo intends to encourage people to live healthier. Included are product reformulation, changes in marketing and related information practices to encourage consumers to make more informed choices, and ways to improve the affordability and accessibility of products in underserved communities. PepsiCo developed the commitments using global nutrition criteria based on recommendations contained in WHO/FAO's Technical Report 916[12], reports of the Institute of Medicine (IOM)[13,14], and the US Dietary Guidelines for Americans.

**Table 2:** PepsiCo's Global Goals and Commitments

Products	Marketplace	Community
Provide more food and beverage choices made with wholesome ingredients that contribute to healthier eating and drinking.	Encourage people to make informed choices and live healthier.	Actively work with global and local partners to help address global nutrition challenges.

▪ Increase the amount of whole grains, fruits, vegetables, nuts, seeds and low-fat dairy in our global product portfolio.	▪ Display calorie count and key nutrients on our food and beverage packaging by 2012.	▪ Invest in our business and research and development to expand our offerings of more affordable, nutritionally-relevant products for underserved and lower-income communities.

▪ Reduce the average amount of sodium per serving in key global food brands by 25 percent by 2015.	▪ Advertise to children less than 12 years of age only products that meet our global science-based nutrition standards.	▪ Expand PepsiCo Foundation and PepsiCo corporate contribution initiatives to promote healthier communities, including enhancing diet and physical activity programs.

▪ Reduce the average amount of saturated fat per serving in key global food brands by 15 percent by 2020.	▪ Eliminate the direct sale of full-sugar soft drinks in primary and secondary schools around the globe by 2012.	▪ Integrate our policies and actions on human health, agriculture and the environment to make sure that they support each other.

▪ Reduce the average amount of added sugar per serving in key global beverage brands by 25 percent by 2020.	▪ Increase the range of foods and beverages that offer solutions for managing calories, like portion sizes.	

* Details are available at http://www.pepsico.com.

PepsiCo's global nutrition criteria build on the concepts of "nutrients to limit" and "nutrients and food groups to encourage," and serve as the practical translation of nutrition science and dietary recommendations for marketers and food and beverage developers. "Nutrients to limit" are those of public health concern when consumed in excess and include reduction efforts on sodium, saturated fat and sugar intake. "Nutrients and food groups to encourage" are based on regional nutritional needs, including those related to micronutrient deficiencies and essential fatty acids requirements. Greater attention is being given to increasing the nutritional quality of the PepsiCo portfolio by expanding product offerings that include nuts, wholegrain, vegetables and fruits.

Looking forward, these global goals and commitments will together form a coherent package of actions that will enable PepsiCo to contribute to addressing nutrition needs. PepsiCo drew upon its actual practices in leading countries to determine implementation details, such as PepsiCo UK's recently released progress report and future plans (Tables 3 and 4). Over the last few years, for example, the level of saturates in Walkers crisps has been reduced by 70 to 80 percent while salt levels have been reduced by 25 to 55 percent across the entire Walker product line. PepsiCo UK's experience in shifting nutrient levels, in delivering innovative and nutritious products, and in changing marketing practices creates a platform for global implementation of the new goals and commitments.

**Table 3:** PepsiCo UK Health Journey - Health journey to date

Reformulation	New healthier products	Acquiring healthier brands
SunSeed oil reduced saturated fat across crisps and snacks by 70-80 percent	Walkers Baked (70 percent less fat)	Tropicana

Reduced salt in Walkers by 20-25 percent	SunBites (wholegrain)	Copella

	Pepsi RAW (small portion, natural)	Quaker

	Planet Lunch and Paw Ridge (healthy ranges for children)	V Water

**Table 4:** PepsiCo UK Key Pledges - Future health commitments

Marketing and Community Engagement	Reformulation and Innovation	Stakeholder Engagement and Public Policy
65 percent of carbonated soft drink sales to be no sugar, by 2015	50 percent of savory snacks to be baked, or include positive nutrition*, by 2015	Work with NGOs, think tanks, and others in the food industry to encourage improved health reporting and transparency

60 percent of total sales (by volume) defined as healthier**, by 2015	Invest 70 percent of R&D budget to deliver products defined as healthier**, from 2012	Engage with government, and other stakeholders, to identify greater R&D support for investment in public health

Deliver 1.8 billion servings of fruits and vegetables, and 1.7 billion servings on wholegrain per year, by 2012	Introduce a single serve cap of 160 calories across savoury snacks without significant positive nutrition*, by 2015	Work with government, and other stakeholders, to deliver pledges on portion sizes and retail availability of healthier products

Encourage wider availability of no-sugar drinks in cinemas, theme parks and pubs, by 2012	4 percent reduction in the sugar level of regular Pepsi by 2012	Quaker and Tropicana will be donated to breakfast clubs in deprived areas, serving 10,000 children every day by 2010

Widen availability of fruit juice in fast food outlets, by 2012	All Walkers crisps and snacks to meet or surpass existing FSA salt reduction targets by 2012	

All UK Pepsi advertising supporting the growth of no-sugar or natural, from 2010		

Trial marketing campaigns to transition consumers who have high per-capita consumption of savoury snacks and full-sugar soft drinks to healthier alternatives, from 2010		

* Contain nutritionally significant amounts of fibre, wholegrain, fruits, vegetables or micronutrients

**Meets the FSA Nutrient Profile model (or other equivalent international standards in future)

Only the commitments related to human sustainability are given here. Complementary goals related to the environment include specific targets for energy and water use, recycling, soil conservation, local sourcing from farmers, and reducing the carbon footprint of products. One goal is shared between the PepsiCo environment and human pillars: "integrating...policies and actions on human health, agriculture and the environment to make sure they support each other." Turning this goal into action involves developing health and environmental impact assessment methods to be applied to new products and processes under development.

## Constraints on making more rapid progress

Many recommendations to food companies regarded as simple have turned out to be complex, requiring deeper insights into the limitations of science, the role of supply chains and commodity prices, farmers, retailers, and of consumer behavior[11]. For instance, there have been calls for food companies to lower the level of saturated fats in the oils they use as a means of reducing cardiovascular disease risks. Implementation of such a call is not easy. With the price of palm oil relatively cheap, customer affordability makes it more difficult to build a case for use of alternative oils with improved fatty acid profiles. Further, the inherent productivity of palm versus sunflower and other oilseeds favors palm. Sudden switches from one oil to another oil in a manufacturing process is unrealistic and in many instances, undesirable. Rather, a well-structured long-term plan is required and includes investing in a range of oils that can meet large scale supply demands, supporting research to reduce the saturated fat levels of commonly used edible oils, reviewing pricing and subsidies for oils, and shifting palm oil use from unsustainable to certified sustainable sources. The recent announcement by Unilever to distance itself from a major palm-oil producer discovered to be clearing protected rainforest is a positive case study of such change[15]. Building a future supply of more suitable oils is especially important in countries like China and India where consumption has soared in recent decades[14].

Food companies face challenges outside of their control that influence their ability to design more food and beverage choices that contribute to healthier eating and drinking. Global environmental changes will affect crop availability: India's weakest monsoon in almost four decades has damaged rice and oilseed crops, while cold weather and drought in China may shrink soybean and corn harvests[16]. These environmental disruptions will impact the cost of commodities. In sub-Saharan Africa, all countries surveyed by FAO reported higher domestic rice prices in 2009 than 2008, while 89 percent reported higher prices for maize, millet, and sorghum[17]. Continued environmental pressures, increased global consumption, and the use of crops such as corn and soybean for alternative fuels will continue to hamper the efforts of food suppliers. There is also concern that the amount of meat consumed in developing countries is growing - in the past year, growth has been three times higher than in developed countries[18]. Meat-based diets require more energy, land, and water resources than vegetarian, meaning that the rise of meat consumption will exacerbate resource scarcity for grain and crop production[19].

A further constraint in improving global nutrition is the lack of capacity in nutrition science. Emerging economies are beset by dual burdens of under- and over-nutrition crises. The human capacity to address these needs is weak, and evident when studying nutrition output from researchers. The proportion of full-length publications in leading science and medical journals (based on citation indexes) by country of the first author, nutrition topic, and year was examined from 1991-2007. For the last 2 years, only about 5% of first authors for any nutrition category were from India or China - two countries that account for 40% of the world's population[20]. This weak public sector nutrition science creates serious obstacles for corporate innovation.

Greater R&D intensity is one route to the disruptive innovation critically needed in the food industry. R&D intensity is a well-established indicator of industry innovation[21]. The pharmaceutical and biotechnology industry has consistently ranked highest for several years by this indicator (spending about 15-20% of sales on R&D), while the food industry is typically among the lowest spenders at 1-2% of sales[22]. Even among government institutions the exact total percentage spent on food-based solutions, while hard to calculate is likely to be small. The National Institutes of Health (NIH) holds the majority of US government research spending on nutrition and obesity at roughly 1.4 billion and 700 million, respectively. They fall short of the levels provided for research related to infectious and emerging infectious diseases, bioengineering, and others[23]. Further, the major outcomes of NIH nutrition and obesity research often lead to new medication or surgical solutions as opposed to sustainable food-based solutions. This mismatch between where R&D resources are spent contrasts with recommendations of a global and diverse set of experts who have identified the top 20 policy and research priorities for chronic diseases[24], a number of which involve food and nutrition policy. A significant increase in publicly financed research into food- and lifestyle-based solutions to chronic diseases would stimulate innovation among private and public researchers and implementers.

### Public calls for food companies to adopt certain standards when implementing self-regulatory systems

Potentially some of the greater challenges facing food companies are the levels of mistrust aimed at corporate entities. Brownell and Warner[25] recently proposed recommendations for responsible corporate food practices. In a related article Sharma et al[26] called for a set of standards to be adopted by food companies as they implement self-regulatory systems. PepsiCo believes that several suggestions made by the authors have merit and should be implemented within food and beverage companies. As Sharma stated, food policies and standards should be science-based and draw upon the findings of major scientific bodies such as IOM in the US and WHO globally. Brownell and Warner[25] are correct in that there is a need for greater transparency with respect to industry funding for and relationships with scientists.

In addition, better codes should be developed for lobbying and advocacy. PepsiCo acknowledges that there will be real differences of opinion between advocates within and outside of industry that should be respected and debated on the basis of their overall public benefit. There are many areas of uncertainty when it comes to developing nutrition policy, which require experimentation and diverse approaches. Nowhere is this truer then with respect to obesity. Scientists and policymakers have yet to find large-scale examples of what works well to reduce obesity at the population level and most clinical studies demonstrate that early weight changes are not sustained beyond a year.

As Brownell et al said, that there is a need "to combine personal and collective responsibility approaches in ways that best serve the public good[27]." The value of self-regulation is especially great in countries with weak to absent government regulatory capacity. Food companies are increasingly making public pledges with respect to reformulation goals, marketing restrictions to children, and labeling. Independent audit bodies should monitor pledges with the results placed in the public domain. For example, the Healthy Weight Commitment Foundation (HWCF) is using the Robert Wood Johnson Foundation (RWJ) as an auditing body. This partnership between industry, non-profits, and educators aims to reduce obesity in the US by 2015 and will have each of its platforms independently evaluated by RWJ[28]. In addition, companies are subject to many independent monitoring schemes that include the Dow Jones Sustainability Index and the Global Reporting Initiative[29]. Their reports to the investor and business community create incentives for positive corporate behaviors while being critical of others not valued by shareholders and long term investors.

## The need for increased private-public collaboration

Achieving product goals requires a considerably increased investment in research, close interaction with those working on agricultural commodities, and deeper insights into how future consumers will respond to healthier products. Some consumers are concerned about having to compromise taste for better health, to pay more, or to give up something to which they are accustomed. Public health has tended to undervalue consumer insights and taste preferences in promoting healthy eating[27].

Questions of consumer preference, as well as examples of complexity mentioned in earlier sections of this article, are not presented as reasons for delay or inaction. Rather they are discussed because of the authors' own experiences in the public health sector (note that GM worked at the CDC, MK the Mayo Clinic, and DY at WHO) that by openly highlighting the roadblocks companies face in delivering a healthier portfolio, colleagues in the public sector will be encouraged to partner in finding solutions.

Recognition of the potential for well-constructed private-public alliances to address chronic disease has been growing over the last year. At the World Economic Forum meeting in Dubai in November 2009, the Global Agenda Council for chronic diseases, which includes representation from the private and public sectors, proposed development of an "Action Coalition" to stimulate joint action and promote policy coherence across sectors and businesses. The coalition will collaborate with WHO's Global Non-communicable Disease (NCD) Network, supporting the implementation of the WHO NCD Action Plan. At the latest meeting of NCD-Net, the WHO Director General and Klaus Schwab, Executive Chairman of the WEF expressed their strongest support for such actions to be expedited.

An IOM 2010 report focused on reducing the global burden of cardiovascular disease stated: "many intervention approaches ...are more likely to succeed if public education and government policies and regulations are complemented by the voluntary collaboration of the private sector[30]." Specifically, it was suggested that the food industry increase international collaborations aiming to reduce unhealthy ingredients while also placing restrictions on marketing of unhealthy products. These recommendations support the creation of more private-public alliances and are in agreement with the steps that food companies are already taking to address chronic disease.

The progress and goals reported here provide a platform for accelerated actions that could have major positive implications for public health.

## Conflicts of interests

DY played a role in steering the consultative process for the CEO meetings under Gro Harlaem Brundtland while then at WHO.

## Authors' contributions

DY; MK; GM led conception and development of the major arguments; SK advanced the work of IFBA versus WHO; RB led development of the UK component; all contributed to final editing and approval
